# Supplementary material for: Quantum spin resonance in engineered proteins for multimodal sensing
Source: Nature. 2026 Jan 21;649(8099):1172–9. doi: 10.1038/s41586-025-09971-3 (PMC12851924; doi:10.1038/s41586-025-09971-3)
Supplement: Supplementary file 2 — Reporting Summary [file 41586_2025_9971_MOESM2_ESM.pdf]

nature portfolio

Corresponding author(s): Harrison Steel

Last updated by author(s): Nov 3, 2025

Reporting Summary

Nature Portfolio wishes to improve the reproducibility of the work that we publish. This form provides structure for consistency and transparency in reporting. For further information on Nature Portfolio policies, see our [Editorial Policies](#) and the [Editorial Policy Checklist](#).

Statistics

For all statistical analyses, confirm that the following items are present in the figure legend, table legend, main text, or Methods section.

n/a	Confirmed	
☐	☒	The exact sample size (*n*) for each experimental group/condition, given as a discrete number and unit of measurement
☐	☒	A statement on whether measurements were taken from distinct samples or whether the same sample was measured repeatedly
☒	☐	The statistical test(s) used AND whether they are one- or two-sided<br>*Only common tests should be described solely by name; describe more complex techniques in the Methods section.*
☒	☐	A description of all covariates tested
☒	☐	A description of any assumptions or corrections, such as tests of normality and adjustment for multiple comparisons
☐	☒	A full description of the statistical parameters including central tendency (e.g. means) or other basic estimates (e.g. regression coefficient) AND variation (e.g. standard deviation) or associated estimates of uncertainty (e.g. confidence intervals)
☒	☐	For null hypothesis testing, the test statistic (e.g. *F*, *t*, *r*) with confidence intervals, effect sizes, degrees of freedom and *P* value noted<br>*Give P values as exact values whenever suitable.*
☒	☐	For Bayesian analysis, information on the choice of priors and Markov chain Monte Carlo settings
☒	☐	For hierarchical and complex designs, identification of the appropriate level for tests and full reporting of outcomes
☒	☐	Estimates of effect sizes (e.g. Cohen's *d*, Pearson's *r*), indicating how they were calculated

Our web collection on [statistics for biologists](#) contains articles on many of the points above.

Software and code

Policy information about [availability of computer code](#)

Data collection	Data collection code used Python (v3.11.11), SciPy (v1.15.1) , NumPy (v.126.4) , scikit-learn (v1.6.1) and scikit-image (v0.20.0) in addition to manufacturer-provided drivers and software for instruments used in the work (instruments identified in methods/supplementary notes).
Data analysis	Data analysis code used the DeLTA library (v2), Python (v3.11.11), SciPy (v1.15.1) , NumPy (v.126.4) , scikit-learn (v1.6.1) and scikit-image (v0.20.0). Code required to generate figures has been uploaded alongside data as per data availability statement below.

For manuscripts utilizing custom algorithms or software that are central to the research but not yet described in published literature, software must be made available to editors and reviewers. We strongly encourage code deposition in a community repository (e.g. GitHub). See the Nature Portfolio [guidelines for submitting code & software](#) for further information.

Data

Policy information about [availability of data](#)

All manuscripts must include a [data availability statement](#). This statement should provide the following information, where applicable:

- Accession codes, unique identifiers, or web links for publicly available datasets
- A description of any restrictions on data availability
- For clinical datasets or third party data, please ensure that the statement adheres to our [policy](#)

The experimental data that support the findings of this study and code required to generate the figures are available at \href{https://doi.org/10.25446/

oxford.30344995}{10.5281/oxford.30344995}. Plasmid sequencing data have been made available on the European Nucleotide Archive (ENA) with the identifier \href{https://www.ebi.ac.uk/ena/browser/view/PRJEB83586}{PRJEB83586}

Research involving human participants, their data, or biological material

Policy information about studies with human participants or human data. See also policy information about sex, gender (identity/presentation), and sexual orientation and race, ethnicity and racism.

Reporting on sex and gender	NA
Reporting on race, ethnicity, or other socially relevant groupings	NA
Population characteristics	NA
Recruitment	NA
Ethics oversight	NA

Note that full information on the approval of the study protocol must also be provided in the manuscript.

Field-specific reporting

Please select the one below that is the best fit for your research. If you are not sure, read the appropriate sections before making your selection.

☒ Life sciences ☐ Behavioural & social sciences ☐ Ecological, evolutionary & environmental sciences

For a reference copy of the document with all sections, see nature.com/documents/nr-reporting-summary-flat.pdf

Life sciences study design

All studies must disclose on these points even when the disclosure is negative.

Sample size	For Figs 1, 2 and 4 sample size was determined by the number of cells in a field of view (~1000) that could be fit while clearly distinguishable. Fig 4 panels H-G was due to the number of cells that were randomly successfully loaded into the mother machine, with a field of view selected to maximise the number of visible cells. Fig 3 was a bulk spectroscopy measurement and therefore sample size of 1 was sufficient. Fig 6 was also bulk measurements, repeated in triplicate for the purpose of eliminating stray photobleaching as an effect, but not for any statistical purpose. For all figures experiments were repeated multiple times as resources and sample availability permitted, and error bars included (specified per-figure in the manuscript). Note that no statistical testing methodology which explicitly depends on numbers of samples was performed in the work.
Data exclusions	Data with the clearest signal-to-noise was chosen for presentation in the final manuscript, not based on pre-established criteria.
Replication	Experiments were repeated 2-3 times on different days starting from fresh growth of bacteria, as time and resources allowed. Experiments were replicated in the same laboratory using the same equipment. Only one iteration of each experiment was included (as above, chosen based on signal-to-noise). All results were successfully reproduced, however we note that microfluidic experiments are highly chip- and setup-dependent therefore reasonable reproduction requires precise adherence to protocol and set-up parameters.
Randomization	Not relevant.
Blinding	Not relevant. All analysis was performed algorithmically with the same code applied to each group in a given experiment.

Reporting for specific materials, systems and methods

We require information from authors about some types of materials, experimental systems and methods used in many studies. Here, indicate whether each material, system or method listed is relevant to your study. If you are not sure if a list item applies to your research, read the appropriate section before selecting a response.

Materials & experimental systems

n/a	Involved in the study
☒	☐ Antibodies
☒	☐ Eukaryotic cell lines
☒	☐ Palaeontology and archaeology
☒	☐ Animals and other organisms
☒	☐ Clinical data
☒	☐ Dual use research of concern
☒	☐ Plants

Methods

n/a	Involved in the study
☒	☐ ChIP-seq
☒	☐ Flow cytometry
☒	☐ MRI-based neuroimaging

Plants

Seed stocks	*Report on the source of all seed stocks or other plant material used. If applicable, state the seed stock centre and catalogue number. If plant specimens were collected from the field, describe the collection location, date and sampling procedures.*
Novel plant genotypes	*Describe the methods by which all novel plant genotypes were produced. This includes those generated by transgenic approaches, gene editing, chemical/radiation-based mutagenesis and hybridization. For transgenic lines, describe the transformation method, the number of independent lines analyzed and the generation upon which experiments were performed. For gene-edited lines, describe the editor used, the endogenous sequence targeted for editing, the targeting guide RNA sequence (if applicable) and how the editor was applied.*
Authentication	*Describe any authentication procedures for each seed stock used or novel genotype generated. Describe any experiments used to assess the effect of a mutation and, where applicable, how potential secondary effects (e.g. second site T-DNA insertions, mosiacism, off-target gene editing) were examined.*
